# Correlation study of brain function changes after chemotherapy in breast cancer patients by automated fiber quantification based on diffusion tensor imaging

**DOI:** 10.1371/journal.pone.0339924

**Published:** 2025-12-31

**Authors:** Yun Feng, Wei Chen, Yang Lu, Haifei Zhou

**Affiliations:** Department of Radiology, The Affiliated Huai’an NO.1 People’s Hospital of Nanjing Medical University, Huai’an, Jiangsu, China; Pisa University Hospital, ITALY

## Abstract

**Purpose:**

The aim of this study is to evaluate the changes of white matter microstructure in breast cancer patients before and after chemotherapy based on automated fiber quantification (AFQ),as well as determine if these dispersion indexes are significantly correlated with clinical data.

**Materials and Methods:**

Twenty-four breast cancer patients scheduled for chemotherapy were enrolled. Diffusion tensor imaging (DTI), neuropsychological tests and self-report measures, and hematological tests were conducted before chemotherapy(time 0,T0) and within one week after chemotherapy(time 1,T1). AFQ was used to track 20 fiber tracts in the brain. The correlation between average abnormal tracts and changes in neuropsychological tests and self-report measures and blood indicators was analyzed.

**Results:**

Compared to T0, subjects at T1 showed decreased scores on the verbal fluency test; increased scores on the self-rating anxiety scale(SAS)and self-rating depression scale (SDS). Estrogen concentration was lower while luteinizing hormone(LH), follicle-stimulating hormone, and triglyceride levels were higher. Mean fractional anisotropy (FA) value decreased in the right cingulum cingulate(CGC)while mean radial diffusivity (RD) increased in the right CGC; mean axial diffusivity (AD) value decreased in callosum forceps major and callosum forceps minor. Changes in FA with in the right CGC were positively correlated with changes in SDS and LH, while changes in RD with in the right CGC were negatively correlated with changes in SDS and LH.

**Conclusion:**

Early changes observed in brain white matter fiber tracts, along with persistent hormone and triglyceride metabolism disorders, could potentially serve as neurobiological markers for monitoring chemotherapy-induced cognitive impairment.

## Introduction

Cancer-related cognitive impairment (CRCI) has been widely reported to affect a wide range (17–78%) of patients, primarily in memory, attention, psychomotor speed, and executive functioning [1].CRCI can occur before the start of treatment, during treatment, and persist for several years after treatment [1–4]. Although the mechanism of CRCI is still controversial, animal models and neuroimaging studies suggest that neurotoxicity caused by chemotherapy drugs may be one of the main factors contributing to cognitive impairment [5]. The long-term quality of life for breast cancer survivors can be affected by chemotherapy-induced neurotoxicity [1]. Therefore, early and accurate diagnosis of CRCI is crucial for guiding clinical preventive management and subsequent functional exercises for patients. Imaging plays an irreplaceable role in diagnosing abnormal brain structure and function associated with cognitive impairments in various diseases. Previous neuroimaging study has shown that breast cancer patients undergoing chemotherapy exhibit disrupted white matter microstructure and impaired structural connectivity, suggesting potential neuropathological mechanisms underlying chemotherapy-induced cognitive impairment. [6].

Diffusion tensor imaging(DTI)is a noninvasive neuroimaging technique commonly employed to evaluate the integrity of white matter microstructure within the central nervous system [7–9]. A range of analytical approaches has been established for DTI analysis, including region of interest (ROI)-based methods, voxel-based analysis (VBA), and tract-based spatial statistics (TBSS), each offering distinct advantages in capturing white matter integrity with varying degrees of anatomical specificity and sensitivity.. However, none of these methods can accurately localize individual fiber tracts [8].Daniel et al. [10] evaluated white matter microstructural changes 5–15 years after chemotherapy in elderly patients with long-term breast cancer based on TBSS and found that fractional anisotropy (FA) decreased over time in the corpus callosum(CC), anterior corona radiata(SCR), and body and genu of the external capsule in the chemotherapy group. Huawen Zhang et al. [11]evaluated whole-brain white matter microstructural alterations in breast cancer survivors based on TBSS and showed significant FA reduction, radial diffusivity (RD) increase, and axial diffusivity (AD) reduction in multiple brain white matter regions compared with healthy controls. An overlap of reduced FA and increased RD was found on the CC body and bilateral SCR, whereas an overlap of reduced FA and increased AD was found on the CC body and right SCR. Deprez et al. [12]showed that breast cancer patients undergoing chemotherapy had lower FA values and higher mean diffusivity (MD) values in frontal and temporal white matter than healthy and cancer controls. Deprez et al. also [13]observed that breast cancer patients had lower FA values and higher MD values in frontal white matter months after chemotherapy than before treatment, and in addition, changes in FA were significantly correlated with changes in performance of attention and verbal memory. A longitudinal study by Menning et al. [14] showed that breast cancer patients who received chemotherapy had lower FA values in the right superior longitudinal fasciculus (SLF) than breast cancer patients who did not receive chemotherapy. Evidence from these previous studies suggests that breast cancer patients undergoing chemotherapy have altered white matter microarchitecture, which may be related to differences in their neurocognitive abilities. Automated Fiber Quantification (AFQ) represents a fully automated approach that enables rapid and reliable identification of major white matter fibers while facilitating further investigation into the anatomical characteristics of fiber dispersion. Consequently, AFQ has found extensive applications in clinical and basic research domains such as aging, and certain mental illnesses [9, 15]. The objective of this study was to examine the impact of AFQ on white matter microstructure in breast cancer survivors before and after chemotherapy while exploring potential significant correlations between diffusion indices and clinical data.

## Materials and methods

### Study subjects

This study collected 24 cases of breast cancer patients who visited the breast and thyroid surgery department from April 15, 2023 to February 15, 2024. MRI, neuropsychological scale tests, and blood tests were completed for these patients before chemotherapy (time 0,T0)and within one week after chemotherapy before chemotherapy(time 1,T1). All participants were right-handed with no history of alcohol or drug use, mental disorders, chronic diseases, brain damage, hearing or vision loss, or other conditions affecting brain function. The chemotherapy regimen for all participants consisted of 4−6 courses lasting approximately 5−6 months. Ethical approval was granted by the the Ethics Committee of the Affiliated Huai’an NO.1 People’s Hospital of Nanjing Medical,ethical approval number is YX-Z-2022-053-01and all participants signed written informed consent forms before the study.

### Neuropsychological assessment

All breast cancer survivors underwent a comprehensive battery of neuropsychological tests at T0 and T1, encompassing the following domains: (1) Digit Symbol Test(DST): After the participants became acquainted with the association between the numerical digits and corresponding symbols, the symbols were accurately inserted beneath their respective digits. The total count of correctly filled characters within a time frame of 90 seconds was tallied, assigning one point for each accurate symbol to assess processing speed; (2) Auditory Verbal Learning Test(AVLT):The examiner presented a list of 12 words to the participants, instructing them to immediately recall and subsequently repeat the words after different time intervals, encompassing short-term, short-delayed, and long-delayed recall. Language proficiency and semantic memory capacity were assessed; (3) Verbal Fluency Test(VFT):Utter a specific set of words as frequently as possible within the designated time frame (1 min) to assess linguistic fluency; (4) Serial Dotting Test(SDT):Prior to the test, participants were instructed to practice aligning two lines of dots within the circle, aiming for optimal central placement. The duration of the entire assessment was recorded and utilized as an indicator for assessing fine motor skills; (5) Number Connection Test A(NCT-A):The task involves the random arrangement of Arabic numerals from 1 to 25 on a sheet of paper, and participants are required to connect them in ascending order using a pen as quickly as possible. The time taken for completion is recorded to assess attentional performance. In addition, the Montreal Cognitive Assessment (MoCA) scale was used to evaluate the overall cognitive function. Anxiety levels were determined using Self-Rating Anxiety Scale(SAS), while depression symptoms were assessed via Self-Rating Depression Scale(SDS).

### Biochemical blood tests

Biochemical blood tests were conducted at T0 and T1, encompassing measurements of red blood cell count, hemoglobin concentration, triglyceride(TG) levels, total cholesterol levels, fasting blood glucose levels, luteinizing hormone(LH), follicle stimulating hormone(FSH), and estrogen(E2). Fasting venous blood samples were collected between 7:00–9:00 am.

### Magnetic resonance image acquisition and data processing

(1) MRI examination method: The same 32-channel 3.0T MRI scanner (Discovery MR 750, GE Healthcare, Milwaukee, USA) was utilized for image acquisition. Participants were instructed to keep their eyes closed and remain awake during the scanning session. High-resolution 3DT1-weighted image parameters are as follows: slice thickness: 1 mm, Repetition time (TR): 8.14 ms, Echo time (TE): 3.17ms, flip Angle: 12°, NEX:1, field of view (FOV):240 × 240mm2, matrix:256 x 256, voxel size: 1x1x1 mm3.DTI data was collected using echo planar imaging sequences with the following scanning parameters: TR = 9154 ms; TE = 55 ms; FOV:224 mm *224 mm; voxel size:2 mm*2 mm*2 mm; collection included a total of thirty-two diffusion weighted directions and took approximately387 seconds. (2) Data preprocessing: FSL5.0 software(http://www.fmrib.ox.ac.uk/fsl/) was employed for AFQ analysis in DTI data preprocessing procedures. First, the Dicom format of the original data was converted to Nifti format. Each diffusion-weighted image was registered to the b = 0 image using the FMRIB Linear Registration Tool (FLIRT) to correct for distortions arising from eddy currents and head motion.Then, the transformation matrix was extracted and the diffusion gradient direction was rotated. Brain masks for the T1-weighted imaging (T1WI) and b = 0 images were generated using the Brain Extraction Tool (BET) with a fractional intensity threshold set at 0.2.The FMRIB diffusion tool (FDT) was used to construct the modified diffusion gradient direction matrix diffusion tensor model, reconstruct the diffusion tensor, and simultaneously generate FA, MD, AD, RD and S0 images. The AFQ software package on MATLAB was used to trace 20 fiber tracts in the brain and quantitate 30 points on each fiber tract: all anatomical images were aligned to the AC-PC plane using the script mrAnatAverageAcpc-Nifti. The script dtiMakeDt6FromFSL was used to align the T1WI image, the anatomical reference, with the S0 image, and dt6 MATLAB format files were obtained for further analysis. Will all diffusion MRI dataset import AFQ package (version 1.2, https://github.com/yeatman-lab/AFQ) in MATLAB R2012b MathWorks. The program returns tensor-based measurements of 100 equidistant line segments along 20 regions an automatic algorithm [16–17]. In particular, the AFQ consists of a three-step procedure:(1) whole-brain tractography, (2) Fiber tract segmentation based on path point ROI [18], and (3) cleaning and refinement of tractography based on probabilistic tractography [19]. The following 20 fibers were traced: bilateral anterior thalamus radiation (ATR), corticospinal tract (CST), cingulum cingulate (CGC), cingulum hippocampus (CGH), inferior fronto-occipital fasciculus (IFOF), inferior longitudinal fasciculus (ILF), superior longitudinal fasciculus (SLF), uncinate fasciculus (UF), arcuate fasciculus (AF), callosum forceps major [posterior forceps of the corpus callosum, CCF_P], and callosum forceps minor [anterior forceps of the corpus callosum, CCF_A].

### Statistical analysis

The SPSS25.0 software was employed to assess the general information and clinical data of all subjects. The Kolmogorov-Smirnov test was conducted to examine the normality of continuous variables.Data conforming to a normal distribution were reported as the means ± standard deviations, whereas data not adhering to a normal distribution were expressed as the medians and interguartile ranges (M (Q1–Q3)). Paired t-tests were utilized for normally distributed data to compare changes from T0 to T1 within each group, while Wilcoxon tests were used instead for non-normally distributed data.Subsequently, Pearson correlation coefficient was employed to statistically examine the associations between the mean abnormal white matter fiber metrics and variations in neuropsychological test scores, self-assessment scale outcomes, and blood-based biomarkers.Statistical significance was considered at a P < 0.05, with P values corrected using false discovery rate(FDR). Due to the high correlation between adjacent points on the path profile, each point should not be regarded as an independent comparison [20], so the point-by-point comparison only retains the results of the differences between adjacent ≧3 nodes.

## Results

### General information

A total of 24 breast cancer survivors, aged between 30 and 65 years with a mean age of 50 ± 9 years, were included in this study. Among them, there were 4 patients with stage I breast cancer, 9 patients with stage II breast cancer, and 11 patients with stage III breast cancer. Ten patients (41.7%) received intravenous administration of TEC regimen consisting of docetaxel (75 mg/m2), epirubicin (50 mg/m2), and cyclophosphamide (500 mg/m2) for six cycles; whereas fourteen patients (58.3%) received intravenous administration of ACT regimen comprising doxorubicin (60 mg/m2), cyclophosphamide (600 mg/m2), and paclitaxel (175 mg/m2) for four cycles.

### The neuropsychological tests and self-report measures and blood biochemical indexes

In comparison to T0, the scores for AVLT exhibited a decrease while SAS and SDS scores demonstrated an increase at T1 (Table 1). There was a significant decrease in E2 concentration along with an increase in LH, FSH, and TG levels at T1 compared to T0 (P < 0.05) (Table 2).

**Table 1:** Neuropsychological tests and self-report measures before and after chemotherapy for breast cancer survivors.

	MOCA (score)	NCT-A(sec)	DST(score)	SDT(sec)	SAS(score)	SDS(score)	VFT(score)	AVLT(sore)
T0	26.1 ± 2.1	51.4(40.3-61.8)	48.0(32.5-58.0)	39.0(31.9-43.6)	28.9 ± 3.7	28.0(24.0-32.0)	8.1(7.5-9.5)	24.0(22.0-27.5)
T1	25.6(24.8-27.0)	42.3(39.4-66.2)	47.0 ± 15.7	36.5(32.1-42.9)	32.5 ± 4.3	33.8 ± 5.8	7.0(5.5-7.5)	19.1 ± 5.9
Z/T	1.353	−0.703	−1.641	0.335	−3.660	−3.989	1.891	4.359
P	0.176	0.482	0.101	0.740	0.001**	<0.001**	0.056	<0.001**

Abbreviations:*MoCA* montreal cognitive assessment,*NCT-A* number connection test A,*DST* digit symbol test,*SDT* serial dotting test,*SAS* self-rating anxiety scale,*SDS* self-rating depression Scale,*VFT* verbal fluency test,*AVLT* auditory verbal learning test.**P < 0.01,*P < 0.05

**Table 2:** Biochemical indices of breast cancer survivors before and after chemotherapy.

	E2(pmol/L)	LH (IU/L)	FSH(IU/L)	WBC (1012/L)	HB(g/L)	GLU (mmol/L)	TC (mmol/L)	TG (mmol/L)
T0	134.0(50.0-348.0)	17.9(4.0-26.2)	18.1(7.7-53.6)	6.6 ± 2.0	123.1 ± 14.0	5.4(4.9-5.9)	5.2 ± 1.2	1.0(0.8-1.5)
T1	71.0(34.5-112.5)	31.2 ± 19.4	65.5(26.9-80.0)	5.5(3.8-7.3)	120.1 ± 9.2	5.0(4.8-5.8)	4.7(4.2-5.4)	2.0(1.5-2.4)
Z/T	−2.876	−3.245	−2.938	−1.150	1.129	−0.854	−1.568	−4.236
P	0.004**	0.001**	0.003**	0.250	0.269	0.393	0.117	<0.001**

Abbreviations*: E2* estrogen,*LH* luteinizing hormone,*FSH* follicle-stimulating hormone,*WBC* red blood cell,*HB* hemoglobin,*GLU* glucose,*TC* total cholesterol,*TG* triglyceride.**P < 0.01,*P < 0.05

### Quantitative analysis of white matter fiber tracts

**Fig 1:**
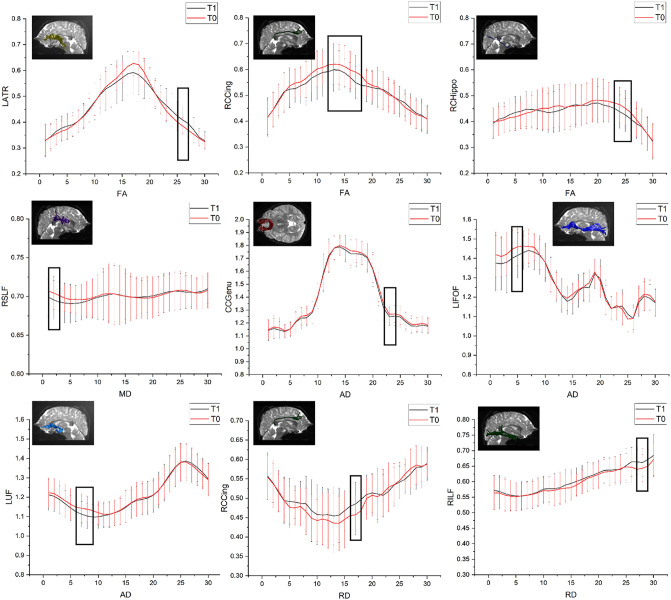
Point-by-point comparison results of each brain white matter fiber tract before and after chemotherapy. The red curve represents T0 (before chemotherapy), while the black curve represents T1 (after chemotherapy). The X-axis indicates the position along the pathway point regions of interest, with parameter names labeled below it. The Y-axis represents DTI values for each parameter, with cerebral white matter fiber tract names indicated next to it.

The mean FA values of the right CGC showed a significant decrease at T1 compared to T0 (FDR corrected, P = 0.001,q = 0.022). Additionally, there was an increase in the mean RD values of the right CGC (FDR corrected, P = 0.002,q = 0.030), and a decrease in the mean AD values of both CCF_P and CCF_A(FDR corrected, P = 0.001,q = 0.017 for CCF_P and P = 0.004,q = 0.035 for CCF_A). The FA value increased from node 25–27 in the left ATR, decreased from node 12–18 in the right CGC, and from node 23–26 in the right CGH. The MD value decreased at nodes 1–3 in the right SLF. AD values decreased at nodes 22–24 in CCF_A, 4–6 in left IFOF, and 6–9 in left UF; The RD value increase from node 13–18 in the right CGC, and from node 27–29 in the right ILF. (FDR corrected, P < 0.05) (Fig 1).

### Correlation analysis

**Fig 2:**
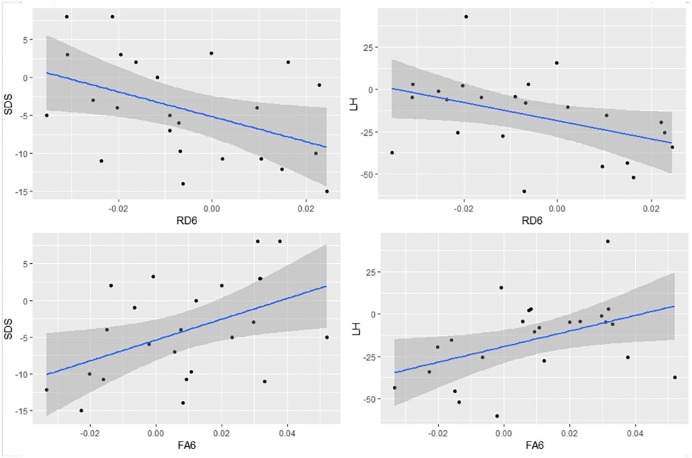
The association between mean abnormal brain white matter tracts and alterations in neuropsychological tests and self-report measures and blood indices. The changes in FA with in the right CGC were positively correlated with changes in SDS (P = 0.022, r = 0.466) and LH (P = 0.032, r = 0.440), while changes in RD with in the right CGC were negatively correlated with changes in SDS (P = 0.030, r = −0.445) and LH (P = 0.041, r = −0.419).

Correlation analysis revealed that changes in FA with in the right CGC were positively correlated with changes in SDS (P = 0.022, r = 0.466) and LH (P = 0.032, r = 0.440), while changes in RD with in the right CGC were negatively correlated with changes in SDS (P = 0.030, r = −0.445) and LH (P = 0.041, r = −0.419). (Fig 2)

## Discussion

AFQ, a relatively novel automated method for quantifying brain white matter fiber tracts, enables the quantification of diffusion parameters at multiple nodes along the fiber tracts [21]. In the present study, we observed a decrease in FA values and an increase in RD values of the right CGC, indicating predominant demyelinating changes in axons. The CGC connects the frontal, parietal, and temporal lobes, forming part of the limbic system along with other regions such as the prefrontal cortex, amygdala, anterior cingulate cortex, hippocampus, and insula [22]. Serving as a central connection between these regions, the CGC plays a crucial role in executive function, emotion regulation and pain perception. Numerous studies [21,23] have highlighted its significance for emotional processing and its contribution to modulating pain perception. Consistent with our findings, previous research [24] has demonstrated that chronic pain conditions like trigeminal neuralgia are associated with negative mood states and anxiety disorders linked to altered CGC activity. Given its inclusion within emotional circuitry framework and contribution to emotion regulation processes. Therefore, it is reasonable to hypothesize that the presence of negative affect, including anxiety and depression, among breast cancer survivors following chemotherapy may potentially exacerbate damage to white matter fiber tracts. Furthermore, Previous study [25] has demonstrated that abnormalities in the cingulate region could contribute to various conditions such as Alzheimer’s disease, schizophrenia, depression, post-traumatic stress disorder (PTSD), and mild cognitive impairment.

DTI reconstructs white matter fiber tracts and characterizes their diffusion properties by detecting the movement of water molecules, thereby enabling the identification and description of microstructural abnormalities in white matter, including alterations in axon morphology, axonal density, myelin sheath integrity, and regional variations in water content. [26]. Currently employed parameters for DTI analysis include FA, MD, AD, and RD. FA provides comprehensive information on white matter fiber density and myelin sheath integrity; a decrease in FA may indicate less compacted white matter fiber tracts [27]. MD reflects the extent of unhindered diffusion of water molecules primarily influenced by myelin sheath integrity; an increase in MD may suggest demyelination or edema within white matter fiber tracts [28]. The AD and RD represent the rates of axial and radial diffusion, respectively. AD quantifies the diffusion rate along the white matter fiber tracts, primarily reflecting axonal degeneration pathology. On the other hand, RD is utilized to measure the diffusion rate perpendicular to the fiber bundle. The RD value primarily reflects myelin sheath integrity, with elevated RD indicating damage or loss of the myelin sheath, which is closely associated with demyelination [28]. Additionally, Brusini et al. [29] discovered that RD can serve as an indicator for changes in myelin content.We found that the changes of FA in the right CGC were positively correlated with the changes of SDS and LH. The change of RD was negatively correlated with the change of SDS and LH.The findings suggest that, within the study sample, greater reductions in white matter fiber integrity in the right CGC are associated with more pronounced depressive symptoms and elevated LH levels following chemotherapy. Similarly, increased markers of myelin injury in the same brain region correlate with the severity of depression and higher post-chemotherapy LH levels. However, due to the limited sample size, the stability of these associations remains uncertain. The observed relationships reflect characteristics specific to the current sample and may not be generalizable to all breast cancer patients undergoing chemotherapy. Therefore, these results should be interpreted as preliminary and require further validation through large-scale, multicenter studies.

The corpus callosum, being the largest connective fiber in the human body, plays a crucial role in memory, motor, and visual functions. Specifically, the callosum forceps minor serves as a bridge between the prefrontal lobes of both hemispheres and is associated with processing speed [30]. In a cross-sectional study utilizing ROI analysis, Abraham et al. [31] demonstrated that DTI can detect chemotherapy-induced changes within the callosum forceps minor that may contribute to cognitive impairment. While this study revealed significant variability in AD values before and after chemotherapy treatment, indicating axonal degeneration, no significant correlation with cognitive impairment was observed. Huang [32] et al., identified the callosum forceps major as an anatomical structure potentially influencing processing speed. This finding is partially supported by Duering et al.‘s identification of this region as a strategic fiber affecting processing speed in patients with cerebral small vessel disease [33].

After undergoing chemotherapy, an increase in FA values was observed in the posterior region of the left ATR, while a decrease in FA values was found in the middle region of the right CGC and the anterior region of the right CGH. The elevation in FA values may indicate a compensatory mechanism for neuronal damage. Furthermore, reduced MD values were specifically located within the anterior part of the right SLF, which is a major fiber tract connecting frontal, parietal, and temporal lobes that plays crucial roles in attention, executive control, visual-spatial cognition, motor process regulation, and language functions [34]. The decline in MD value suggests diminished efficiency of connectivity between relevant brain regions following fiber damage. Additionally, decreased AD values were identified within nodes 22–24 of the left CCF_A, as well as within the posterior segment of both IFO and temporal lobe portion of the left UF. The UF belongs to a white matter tract associated with frontal-limbic circuitry that connects lateral orbitofrontal cortex and prefrontal cortex to anterior temporal lobe and basolateral amygdala [35]. The UF plays a crucial role in memory and cognition [36]. The IFOF is a large cortico-cortical association tract that connects the orbitofrontal, posterior temporal, and occipital regions. Importantly, the left IFOF has been recognized as a ventral pathway involved in semantic processing and executive function [37]. Point-level analysis proves to be more sensitive than white matter fiber tract analysis, enabling detection of microstructural impairments that affect clinical cognition. We propose that impairments at different white matter sites may mediate dysfunction across various cognitive domains. Another intriguing finding was that all nodes showing significant differences after chemotherapy exhibited reduced AD values compared to pre-chemotherapy levels; these nodes were exclusively located within the left hemisphere, suggesting cerebral white matter atrophy [38].

This study revealed no significant difference in the overall cognitive ability of breast cancer survivors pre- and post-chemotherapy; however, both short-term and long-term memory abilities decreased while anxiety and depression levels increased following chemotherapy treatment. Chemotherapy-induced cognitive impairment is typically mild and generally comparable to normal levels. Nevertheless, prolonged treatment may impact cognitive deficits with variations observed in processing speed, executive function, and working memory; indicating that chemotherapy affects the neuropsychiatric system of breast cancer patients. These findings are consistent with previous study [39]. Previous studies have reported an elevation in anxiety and depression among breast cancer patients following chemotherapy [40]. These psychological conditions can adversely impact attention, problem-solving abilities, and memory, which is consistent with the findings of our study. It should be noted that while most chemotherapeutic agents are unable to penetrate the blood-brain barrier, nearly all commonly employed chemotherapeutic agents possess the potential to induce damage to the central nervous system. Furthermore, it is important to acknowledge that patients’ own apprehension and unease towards their illness may also exert an influence on neuropsychiatric scores.

Our study findings demonstrate a reduction in E2 concentration and an elevation in LH, FSH, and TG levels subsequent to chemotherapy treatment, indicating alterations in blood biochemical indices. Previous research has emphasized the significant impact of E2 on brain function, suggesting its potential neuroprotective properties [41]. Fundamental investigations propose that E2 may exert neuroprotective effects by binding to regulatory molecules of the E2 receptor and enhancing choline acetyltransferase concentration [42]. Moreover, fluctuations in E2 levels can readily precipitate mood disorders [43]. We hypothesize that chemotherapy-induced disruption of hormone levels exacerbates symptoms of depression or anxiety among patients. Breast cancer patients also demonstrate long-lasting disruptions in TG metabolism attributed to reduced lipid utilization caused by chemotherapy-driven tumor cell growth, leading to heightened post-treatment lipid levels. We suggest that chemotherapy-related cognitive dysfunction involves multiple factors encompassing physiological, pathological and psychological changes. Henceforth, modifications in white matter fiber tracts along with continual decrease in E2 concentrations and increased TG could potentially predict the onset of acute cognitive impairment following chemotherapy treatment. Furthermore, this study identified raised LH and FSH levels likely resulting from negative feedback triggered by diminished E2 secretion. Previous studies have reported an association between elevated levels of LH and FSH and depression [44]. Furthermore, Freeman [45] demonstrated a significant correlation between depressive symptoms and increased variability in FSH and estradiol levels.

There are several limitations to this study. Firstly, our sample size was relatively small, and despite the significance of these findings.The insufficient sample size directly undermines the statistical power of the analysis, thereby limiting the study’s ability to detect true associations between variables. This limitation increases the risk of false negative findings—namely, the failure to identify actual group differences or meaningful correlations. Additionally, a small sample size results in wider confidence intervals, reflecting reduced precision in effect estimation. Furthermore, limited sample sizes are associated with greater random variability, making it difficult to control for individual-level differences that may confound the outcomes. Moreover, as all participants were recruited from a single center, the combination of this sampling approach and the small sample size may introduce selection bias, further compromising the generalizability and robustness of the findings. To address these limitations, future work will focus on expanding the sample size through multi-center collaboration and extended recruitment periods, with the aim of enhancing statistical power and improving the reliability and validity of the results..As an automated analysis tool based on DTI, AFQ has significant advantages in the analysis of long and clear white matter tracts (such as corticospinal tract, optic radiation, and superior longitudinal fasciculus). However, its technical principle determined its poor suitability for regions with highly complex fiber structure, which may affect the completeness and accuracy of the study results. Another limitation of AFQ is that fiber tracts may not accurately represent true brain information transfer pathways due to constraints in imaging parameters (e.g., turning angle and stopping criteria) [46]. In future research, more advanced algorithms will be introduced to enhance the accuracy of AFQ measurements. The study relied solely on a longitudinal design without incorporating a control group of breast cancer patients who did not receive chemotherapy. This limitation precludes the ability to disentangle the potential effects of “breast cancer disease status” from those of chemotherapy on brain function, thereby making it difficult to ascertain whether the observed changes in brain function are directly attributable to chemotherapeutic treatment. From a clinical mechanistic standpoint, breast cancer, as a malignant neoplasm, may influence brain function through various biological pathways, including systemic inflammation, metabolic disturbances, and neuroendocrine alterations. Furthermore, the lack of a healthy control group hinders the establishment of a normative reference for typical brain function, limiting our capacity to determine whether the observed alterations exceed the “normal physiological range” or stem from pre-existing “baseline differences between breast cancer patients and healthy individuals.” Addressing these limitations will constitute key directions for future research.Assessments were conducted one week following the completion of chemotherapy. However, the impact of chemotherapy on brain function may manifest in two distinct patterns: “acute short-term reactions” and “long-term persistent damage.” Short-term assessments are capable of capturing only the former, while failing to detect the latter. To address this limitation, future studies will incorporate multi-time-point longitudinal evaluations to better capture the dynamic trajectory of cognitive changes.

## Conclusion

Employing the AFQ technique for analyzing DTI data of breast cancer survivors pre- and post-chemotherapy revealed specific fiber tracts and nodes that exhibited damage, which were associated with certain clinical outcomes. Importantly, this comprehensive quantitative investigation of fiber tract damage provided more intricate insights into alterations within these tracts. Early changes in brain white matter fiber tracts, persistent disturbances in E2 and TG metabolism may serve as neurobiological markers for monitoring chemotherapy-related cognitive impairment.
